# Study on the Changes of Structures and Properties of PAN Fibers during the Cyclic Reaction in Supercritical Carbon Dioxide

**DOI:** 10.3390/polym11030402

**Published:** 2019-03-01

**Authors:** Mengmeng Qiao, Haijuan Kong, Xiaoma Ding, Zhifeng Hu, Luwei Zhang, Yuanzhi Cao, Muhuo Yu

**Affiliations:** 1 State Key Laboratory for Modification of Chemical Fibers and Polymer Materials, College of Materials Science and Engineering, Donghua University, Shanghai 201620, China; 2160320@mail.dhu.edu.cn (M.Q.); 1159124@mail.dhu.edu.cn (X.D.); 2160226@mail.dhu.edu.cn (Z.H.); 2170293@mail.dhu.edu.cn (L.Z.); 2180410@mail.dhu.edu.cn (Y.C.); 2 School of Materials Engineer, Shanghai University of Engineer Science, Shanghai 201620, China

**Keywords:** polyacrylonitrile fibers, supercritical carbon dioxide, thermal pre-oxidation, cyclization reaction

## Abstract

Thermal pre-oxidation of polyacrylonitrile (PAN) fibers is a time-consuming and energy-consuming step in the production of PAN-based carbon fibers. In this paper, the effect of temperature on the structures and properties of PAN fibers cyclized in the supercritical carbon dioxide (Sc-CO_2_) medium was studied. The thermal behaviors of the PAN fibers were investigated by Fourier transform infrared spectra (FT-IR), X-ray diffraction (XRD), differential scanning calorimeter (DSC) and thermogravimetric analysis (TGA). The cyclization reaction was sensitive to the heating temperature and gas atmosphere. The FT-IR results of the PAN fibers treated in the Sc-CO_2_ confirmed that the degree of cyclization increased with the increase of the cyclization temperature. Compared with the PAN fibers treated in the air, the PAN fibers treated in the Sc-CO_2_ showed a higher degree of cyclization even at the same temperature. These findings might be related to the osmotic action of Sc-CO_2_ causing the fibers to be further arranged in a regular manner, which was favorable for the cyclization reaction. Moreover, as one kind of high diffusion and high heat transfer media, the heat release during the cyclization of PAN fibers could be quickly removed by Sc-CO_2_, which achieved the progress of the rapid-entry cyclization reaction.

## 1. Introduction

Carbon fibers (CFs), as one of the new materials of lightweight high-performance fibers for composites, are defined as fibers containing at least 92 wt % carbon [1,2]. Owing to the advantages of excellent tensile properties, low densities, and high thermal and chemical stabilities, CFs are widely used in various fields [3,4,5,6,7]. CFs are produced from many different precursors, such as polyacrylonitrile (PAN), mesophase pitch, rayon, etc. [8,9,10], among which PAN-based CFs are the preferred reinforcements for structural composites owing to their excellent strength and stiffness combined with the fact they are lightweight and cost less. The majority of CFs (about 95%) are made from PAN fibers [11,12]. However, PAN-based CFs are not easy to commercialize due to the time-consuming pre-oxidation step, which will significantly increase the manufacturing cost. Therefore, the advanced processing technologies that aim to reduce the production cost of CFs should be developed.

Different methods have been studied to accelerate the pre-oxidation rate of PAN fibers, such as ultraviolet (UV) and gamma rays. Marlon et al. [13] added the addition of one kind of photo-initiator during the spinning process and then induced cyclization and crosslinks in PAN fibers with UV at low temperatures. However, excessive radiation could cause defects in the fibers and it was not easy to control the dose of radiation. Zhao et al. [14] studied the treatment of PAN fibers with different pre-oxidation degrees by gamma rays, followed by carbonization. However, it was necessary to accurately control the amount of gamma radiation. Zhao et al. [15] carried out pre-oxidation experiments over different temperature ranges. This method could obtain CFs with higher mechanical properties but the pre-oxidation time was too long. Lin Jianhua et al. [16] conducted the pre-oxidation experiment of PAN fibers with microwave assistance. However, the cost of the microwave equipment was relatively high, and the speed of pre-oxidation was not well controlled.

The thermal pre-oxidation process was one of the most time-consuming and energy-consuming steps [17,18]. In recent years, great interest has been given to the dyeing of synthetic fibers in Sc-CO_2_ with the aim to achieve their eco-friendly production by manipulating the dyeing temperature, pressure, and time [19,20,21]. During the supercritical dyeing process, Sc-CO_2_ fluid near or slightly above its critical points (T_c_ = 31.10 °C, P_c_ = 7.38 MPa) produced substantial polymer swelling because of the dissolution of CO_2_ in polymers [18]. Therefore, Sc-CO_2_ was used as a non-toxic solvent in extractions [22,23], separations [24], chemical reactions [25], and various other applications [26,27]. Furthermore, CO_2_ gives superior heat transfer properties for near-critical operation. From Table 1, we can obtain the thermal conductivity (λ) of Sc-CO_2_ and air under different conditions, from which we can observe that the λ of Sc-CO_2_ is significantly higher than that of the air, indicating that Sc-CO_2_ can be used as a good heat transfer medium to transfer the heat released during the cyclization reaction.

**Figure 1:**
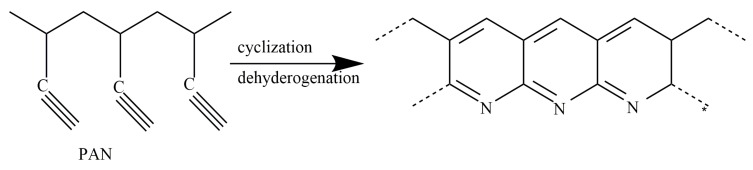
Schematic diagram of cyclization and dehydrogenation of polyacrylonitrile (PAN) fibers.

This study aims to find a new way to use Sc-CO_2_ as a kind of medium to quickly remove the heat released during the cyclization to accelerate the progress of the cyclization and reduce the production cost of CFs. We can see the structural changes of PAN fibers during the cyclization and dehydrogenation from Figure 1. 

## 2. Materials and Methods

### 2.1. Materials and Sample Preparation

PAN fibers (24,000 filaments per fiber bundle) were wet-spun from a copolymer of acrylonitrile/acrylamide/methyl acrylate and supplied by Weihai Fiber Development Co., Ltd., Weihai, China. Carbon dioxide with a purity of 99.99% was supplied by Sinopharm Chemical Reagent Co., Ltd., Shanghai, China.

The reactor was heated to a certain temperature, and then the PAN fibers were fixed on a stirrer in the reaction vessel. The Sc-CO_2_ reactor was closed, the air in the reactor was removed with CO_2_, and then the CO_2_ was passed into the reactor and reached a certain pressure. After a period of reaction, the CO_2_ was removed, the fibers were taken out, and the pre-oxidized fibers we needed were obtained.

### 2.2. Characterizations

Fourier transform infrared spectra (FT-IR) was recorded on the Nicolet 6700 FT-IR spectrophotometer ((Thermo Fisher Company, New York, NY, USA) using the method of KBr troches with a scanning wavenumber range of 400–4000 cm^−1^. The relative cyclization rate (RCR) of PAN fibers was calculated according to Equation (1):

(1) $$RCR=\frac{I_{C=N}}{I_{C=N}+I_{C\equiv N}}\times 100\%$$

where *I*_C=N_: transmission peak intensity of C=N; *I*_C≡N_: transmission peak intensity of C≡N.

X-ray diffraction (XRD) studies of the as-received fibers and pre-oxidized PAN fiber bundles were carried out on the Bruker D/max 2550 VB (Bruker Co., Kanagawa, Japan). The different pre-oxidation fibers and the as-received were all cut into powders before testing using XRD. The range of the scan angle was from 5° to 60° with a step width of 0.02. The aromatic index (AI) of PAN fibers was calculated according to Equation (2):

(2) $$AI=\frac{I_{c}}{I_{c}+I_{p}}\times 100\%$$

where *I*_c_: diffraction intensity of PAN fibers at 2θ = 29°, *I*_p_: diffraction intensity of PAN fibers at 2θ = 16.5°.

Thermal behaviors of pre-oxidation fibers and the as-received fibers were studied using a differential scanning calorimeter (DSC Q100, TA Instrument, Shanghai, China) using a heating rate of 10 °C·min^−1^ in a nitrogen atmosphere; the temperature was varied from 30 to 400 °C. The cyclization degree (CD) of PAN fibers was calculated according to Equation (3):

(3) $$CD=\frac{H_{U}-H_{0}}{H_{U}}\times 100\%$$

where *H*_U_: heat released from as received fibers, *H*_0_: heat released from PAN fibers treated in different temperatures.

The thermal stability of the fibers was investigated by the Thermogravimetric analysis (TGA) (TG 209 F1 Netzsh, Shanghai, China). The ramp rate was 10 °C·min^−1^, and the curves were recorded under a working N_2_ flux equal to 50 mL·min^−1^. The temperature range was employed from room temperature to 900 °C during the test.

The changes in the density of the PAN fibers treated in different conditions were measured by a liquid weight balance (PZ-B-5, Shanghai, China) using the floatation method. After drying, the PAN fibers and the as-received fibers were cut into 0.5 mm or less, and then these were placed in the formulated equilibrium solution which was a mixture of carbon tetrachloride and N-heptane. The proportions of the heavy liquid and the light liquid were adjusted to suspend the fibers until the fibers were evenly distributed in the mixture and remained suspended for more than 4 h. The densitometer was used to determine the density of solution, which was also the density of the PAN fibers.

## 3. Results and Discussion

### 3.1. FT-IR Evolution Analysis of the PAN Fibers Heated at Different Temperatures

**Figure 2:**
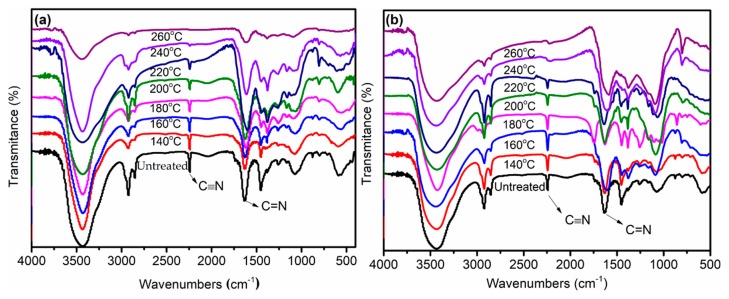
Fourier transform infrared spectra (FT-IR) spectra of PAN fibers treated at different temperatures: (**a**) in Sc-CO_2_; (**b**) in air.

It was suggested that both the shrinkage and decrease in the nitrile group concentration during the pre-oxidation process could be used to monitor the process of pre-oxidation. Figure 2 showed the changes of the infrared spectra of PAN fibers heated at different temperatures in the air and Sc-CO_2_. The peak at 2240 cm^−1^ in the infrared spectra was assigned to the –C≡N stretching of the acrylonitrile unit in the polymer chains. The other absorption peaks in the polymer were given as follows: The double bond stretching vibration zone (1690–1500 cm^−1^), C–H stretching vibration zone (1475–1000 cm^−1^). Figure 2a showed the spectra of as-received and treated PAN fibers in Sc-CO_2_. Comparing the untreated PAN fibers with pre-oxidized PAN fibers treated in the Sc-CO_2_, the absorption at 2240 cm^−1^ showed a reduction for PAN fibers treated at 140 °C in Sc-CO_2_, matching with an increase in the intensity of the absorption band at 1600 cm^−1^. The results showed that the –C≡N bond of the PAN fibers decreased after the heat treatment, and the intensity of the vibrational peak of C=N increased, which also indicated that the ring structures in PAN fibers have been produced after treatment at 140 °C. Further information was also given in the FT-IR spectra: The intensity of the vibrational peak at 1453 cm^−1^ (C–H_2_) gradually weakened, and the intensity of the vibrational peak at 1377 cm^−1^ (C–H) gradually increased. All the changes in the FT-IR spectra indicated that more complex structures have been formed during the cyclization reaction process. Comparing the infrared spectra of PAN fibers treated at different temperatures, with the increase of treatment temperature, the stretching vibration peaks at 1600 and 1377 cm^−1^ gradually increased, while the stretching vibration peak at 2240 and 1453 cm^−1^ gradually weakened. These results indicated that the crosslinked and cyclized structures in PAN fibers increased gradually with the increase of temperature, and accordingly, the degree of cyclization increased with the increase of the cyclization temperature. 

**Figure 3:**
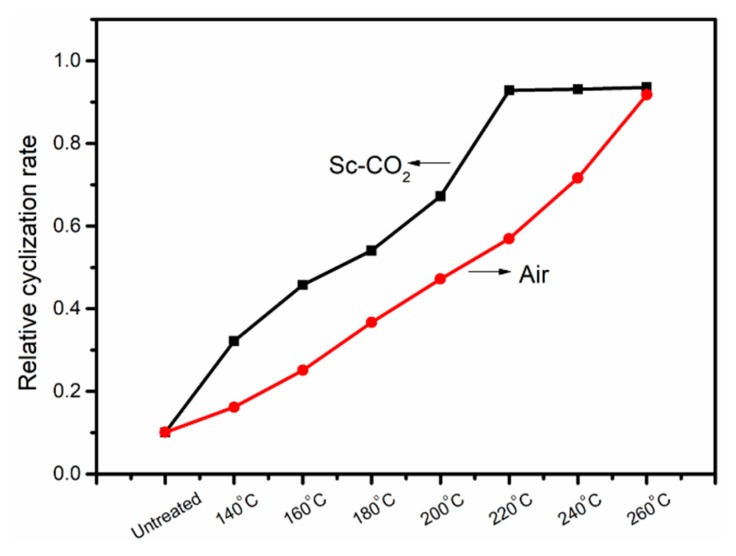
The relative cyclization rate (RCR) of PAN fibers calculated by FT-IR spectra.

The effect of temperature on the cyclization reaction in the air was consistent with the trend in Sc-CO_2_. Comparing the two FT-IR spectra in Figure 2, the changes of absorption spectra at 2240 and 1600 cm^−1^ were more obvious in Sc-CO_2_, indicating that the pre-oxidized PAN fibers treated in Sc-CO_2_ showed a higher degree of cyclization than the PAN fibers obtained in the air, even at the same temperature. The RCR could be calculated using Equation (1). The RCR of fibers treated at different temperatures shown in Figure 3 also indicated that the PAN fibers had a higher degree of cyclization treated in Sc-CO_2_ than the PAN fibers treated in air. It might be due to the fact that Sc-CO_2_, as a kind of fluid, was more likely to carry away the heat released by the cyclization of PAN fibers, thereby accelerating the cyclization of PAN fibers.

### 3.2. X-ray Diffraction Analysis of PAN Fibers Heated at Different Temperatures

**Figure 4:**
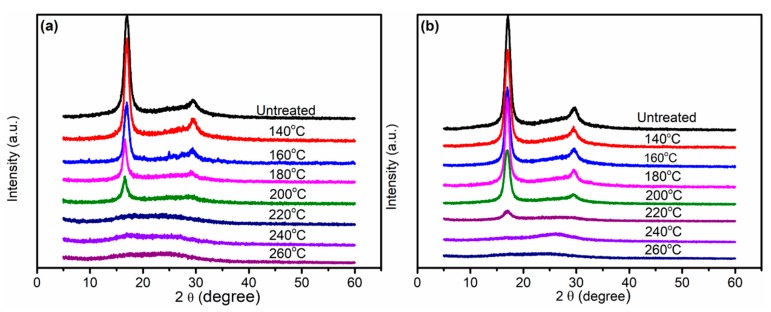
X-ray diffraction (XRD) patterns of PAN fibers treated at different temperatures: (**a**) in Sc-CO_2_; (**b**) in air.

Figure 4 depicts the XRD patterns of PAN fibers treated at different temperatures in air and Sc-CO_2_. The XRD patterns showed the typical diffraction peaks at 2θ = 16.5° and 2θ = 29.0° of PAN fibers, reflecting the ordered crystal structure made up of PAN linear macromolecules. There was a strong diffraction peak at 2θ = 16.5° corresponding to the crystal plane (100), which reflected the spacing of the molecular chains. In addition, there was another weak diffraction peak near 2θ = 29.0° corresponding to the crystal plane (110), which reflected the distance between nearly parallel molecular pieces. A wide diffuse reflection region existed between the two diffraction peaks at 2θ = 16.5° and 29.0°, indicating that the disordered phase was distributed throughout the structure in a non-discrete manner. Due to the cyclization, the structure of as-received fibers was very different from the pre-oxidized fibers; therefore, we could judge the degree of cyclization by the differences in the XRD spectra. As can be seen from the XRD patterns in Figure 4, the diffraction intensity *I*_p_ of the peak at 2θ = 16.5° evidently decreased with the increase of heating temperature, matching with the decrease of the characteristic diffraction peak intensity *I*_c_ at 2θ = 29.0°. When the temperature was 220 °C, the diffraction peak had almost disappeared, which indicated the crystal structure was basically no longer present. This experimental result was consistent with the reduction and conjugation of the C≡N group, which had a predominant role in the change of the PAN molecule during the thermal pre-oxidation. This indicated that the molecular chains in PAN fibers tended to be disordered, which tended to reduce the difference in the ordered regularity between disordered and ordered intervals in the PAN two-phase structure proposed by Lin et al. [33]. These phenomena meant that C≡N in the PAN molecule had reacted to produce C=N, which implied that cyclization and dehydrogenation started to occur in the PAN fibers. At the same time, the higher the treatment temperature, the higher the cyclization reaction degree.

**Figure 5:**
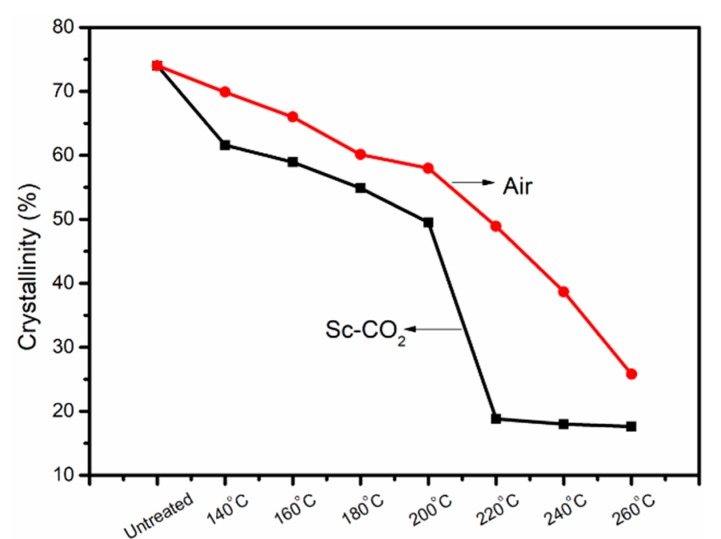
The crystallinities of PAN fibers treated in the air and Sc-CO_2_.

**Figure 6:**
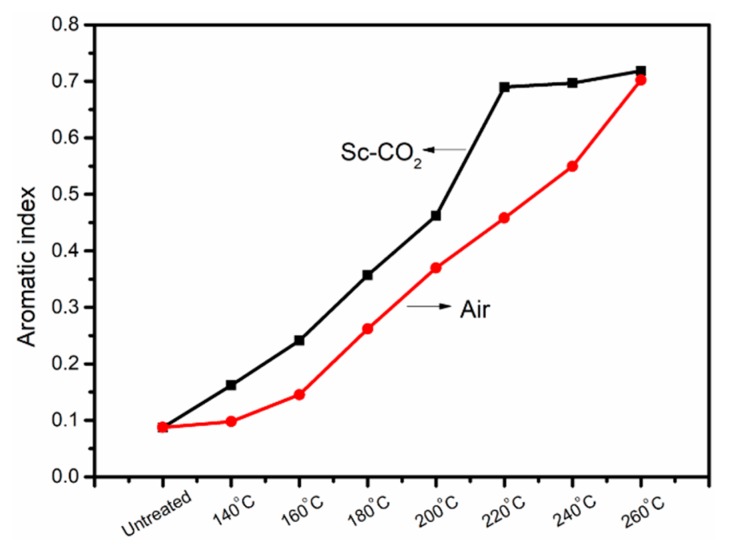
Aromatic Index of PAN fibers treated at different temperatures in air and Sc-CO_2_.

The cyclization of as-received fibers was a reaction that destroyed orientation and decreased crystallinity, which was closely related to the crystallinity. The crystallinity of PAN fibers is given in Figure 5, from which we can observe that the crystallinity of PAN fibers treated in Sc-CO_2_ was greatly reduced, that is to say, a large number of cross-network structures were produced. According to Equation (2), we could calculate the aromatic index (AI) of different pre-oxidation fibers, as shown in Figure 6. The AI of PAN fibers increased with the increase of experimental temperature, at the same time, the fibers treated in Sc-CO_2_ had more cyclized structures than the fibers treated in air, even at the same temperature. These results showed that Sc-CO_2_ fluid was useful to transfer the heat produced during the cyclization reaction and reduce the cyclization temperature to get PAN fibers with high cyclization.

### 3.3. Differential Scanning Calorimeter Analysis of the PAN Fibers Heated at Different Temperatures

**Figure 7:**
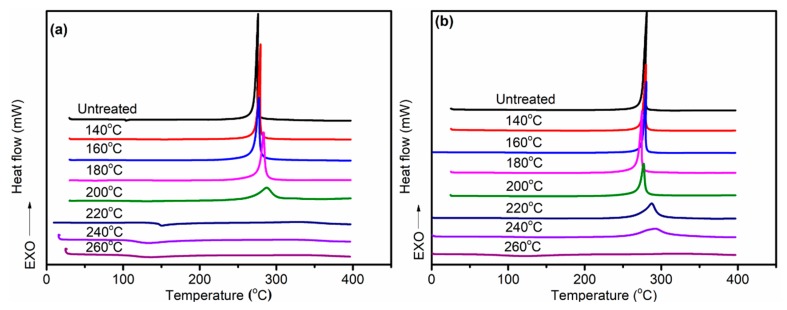
Differential scanning calorimetry (DSC) curves of PAN fibers treated at different temperatures: (**a**) in Sc-CO_2_; (**b**) in air.

**Figure 8:**
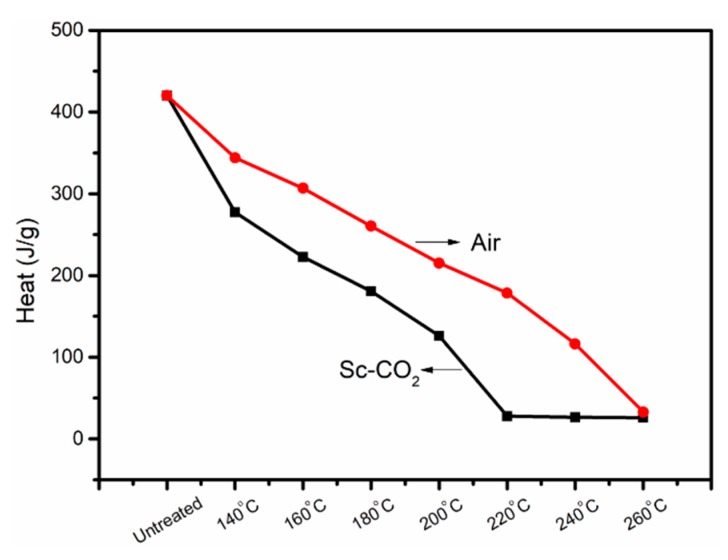
Exotherm of PAN fibers treated at different temperatures in air and Sc-SO_2_.

Figure 7 displays the DSC curves of PAN fibers treated at different temperatures in air and Sc-CO_2_. The effect of heat temperatures on the degree of cyclization was mainly studied by DSC owing to the exothermic process during the cyclization reaction of PAN fibers. Due to the heat resistance of the ring structure, the cyclic structure of the PAN fibers hardly resulted in an exothermic reaction during the heating process of the PAN fibers, while the uncyclized structure underwent an exothermic reaction easily. Therefore, the degree of cyclization of PAN fibers could be calculated from the area of the exothermic peak during the heating process. As can be seen from Figure 7a and Figure 8, there was no obvious change in the area of the exothermic peak treated at 140 °C, but the area of the exothermic peak decreased gradually with the increase of the heat treatment temperature. The total exotherm amount of fibers gradually decreased, indicating that with the increase of the pre-oxidation temperature, the degree of cyclization increased and the heat release of the reaction decreased. At 220 °C, the peak area of the treated fibers clearly decreased, that is to say, the heat release decreased significantly, which indicated that the fibers had a high degree of cyclization. It could also be concluded that, as the temperature increased, the content of exotherm during heating gradually decreased and quantity of the aromatic cyclization structure increased.

**Figure 9:**
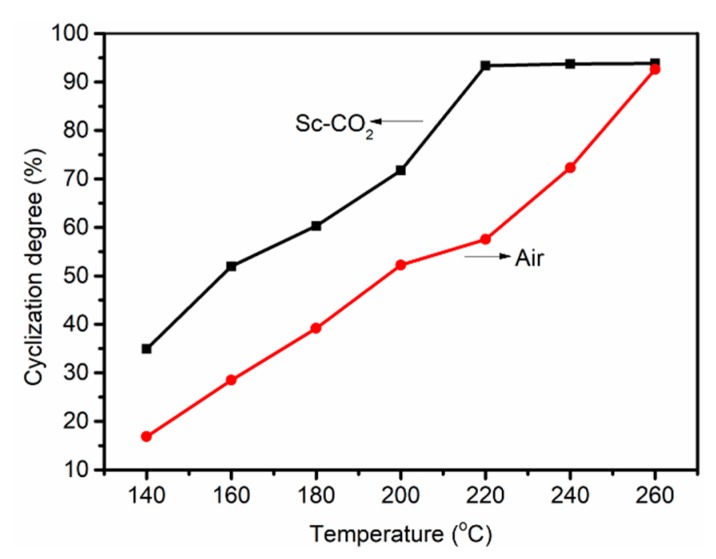
Cyclization degree of PAN fibers treated at different temperatures in air and Sc-SO_2_.

Compared with DSC curves of fibers treated in air in Figure 7b, the exothermic peak of the fibers treated in Sc-CO_2_ was significantly smaller even at the same temperature; therefore, it can be concluded that the fibers treated in Sc-CO_2_ released less heat and had more aromatic cyclization structures. The CD of different PAN fibers was calculated according to Equation (3), as shown in Figure 9. We could interpret the results of experiments in the following sections: The cyclization reaction of PAN fibers was an exothermic process, so the heat released during the cyclization reaction should be taken away in time to prevent the fibers from being fused. The fibers treated in Sc-CO_2_ had smaller exothermic peaks and more cyclized structures compared with the fibers treated in air at the same temperature. This was because Sc-CO_2_ could enter into the inside of the fibers and the heat released by the PAN fibers during the cyclization was taken out in time to speed up the rate of reaction.

### 3.4. Thermogravimetric Analysis of PAN Polymers Heated at Different Temperatures

**Figure 10:**
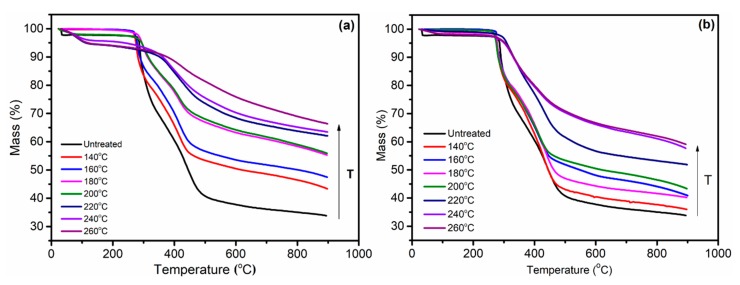
The thermogravimetric (TG) curves of PAN fibers treated at different temperatures: (**a**) in Sc-CO_2_; (**b**) in air.

**Figure 11:**
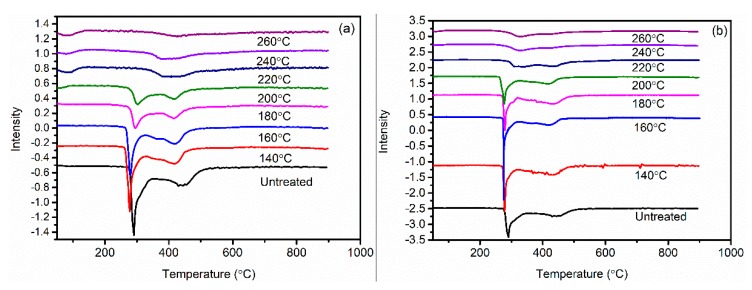
The differential thermal gravity (DTG) curves of PAN fibers treated at different temperatures: (**a**) in Sc-CO_2_; (**b**) in air.

The thermogravimetric (TG) curves and differential thermal gravity (DTG) curves of PAN fibers treated at different temperatures are shown in Figure 10 and Figure 11, respectively. Due to the heat resistance of the ring structure, the cyclic structure of the PAN fibers hardly decomposed during the heating process of the PAN fibers, while the uncyclized structure was more prone to pyrolysis. When the heating temperature was constant, more heat-resistant aromatic cyclization structures were present in PAN fibers, the more difficult the thermal decomposition of fibers became, and the more thermal decomposition residue was obtained. Therefore, it was possible to obtain the degree of cyclization of the PAN fibers by the residual amount of the fibers after the decomposition. The mass of the fibers decreased at the beginning, resulting from the loss of moisture in the fibers. Comparing the pyrolytic residuals and decomposition curves of PAN fibers treated at different temperatures in Table 2 and Figure 10, it could be concluded that as the cyclization temperature increased, the quality of the fibers obtained was increased, which indicated that the amount of the ring structure resistant to decomposition in the cyclized fiber was increased with the increasing temperature. DTG curves showed that there were two decomposition peaks during the heat treatment process. Among them, the weight loss rate was the fastest in the 270–300 °C temperature range. From the DTG curve, we concluded that with the increase of the pre-oxidation temperature, the weight loss rate of the fibers in both temperature ranges is evidently slower, indicating that the content of the ring structures in the fiber was gradually increasing.

At the same time, comparing the residual mass of the fibers obtained in the air with Sc-CO_2_ at the same temperature, it could be concluded that the PAN fibers treated in the medium of Sc-CO_2_ had a greater residual quality than that in air, indicating a higher cyclization structure in PAN fibers treated in Sc-CO_2_. This further confirmed the correctness of the experimental results of the previous FT-IR, XRD, and DSC. When the treatment temperature was 260 °C, the residual mass of PAN fibers treated in Sc-CO_2_ was 66.34%, which was much larger than the residual mass of 61.59% of PAN fibers treated in air, which indicated that the cyclization reaction in Sc-CO_2_ might be a method to improve the carbon yield of CFs.

### 3.5. Density Changes of PAN Fibers Treated in Different Conditions

**Figure 12:**
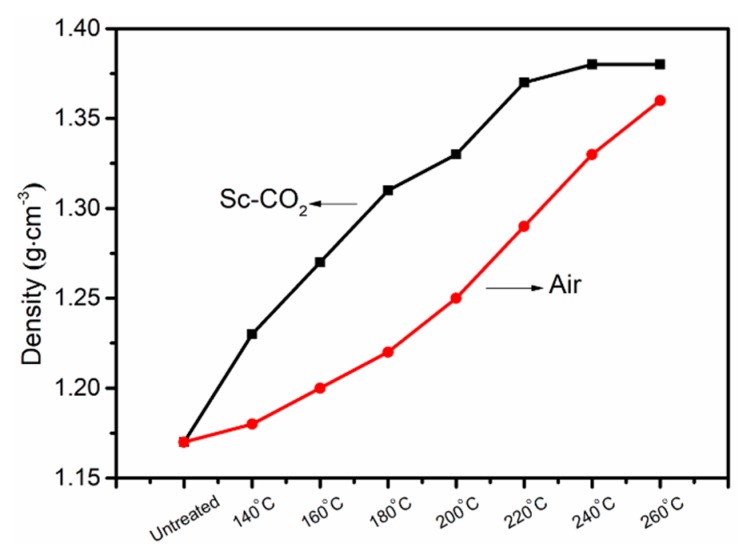
The density changes of PAN fibers.

The density changes of pre-oxidized PAN fibers obtained at different temperatures are shown in Figure 12. The degree of cyclization and densities of PAN fibers had a close relationship owing to the following two reasons. The first reason was due to the effect of drawing to make its physical structure dense, the second was due to the occurrence of the cyclization and cross-linking reaction to increase the density. That is to say, the higher the density of PAN fibers, the higher the degree of cyclization in PAN fibers. It could be seen that as the temperature increased, the density of PAN fibers increased, which was consistent with the increase of the degree of cyclization calculated by the results of FT-IR, DSC, TGA, and XRD, indicating that the degree of cyclization constantly increased with the increasing temperature. Comparing the density of pre-oxidized PAN fibers treated in air with that of those treated in Sc-CO_2_, we could obtain that the degree of cyclization of PAN fibers treated in Sc-CO_2_ was higher than that in air at the same temperature.

It might be related to the osmotic action of Sc-CO_2_. On the one hand, the action of Sc-CO_2_ caused the fibers to be further arranged in a regular manner, which was favorable for the cyclization reaction. On the other hand, Sc-CO_2_ is non-polar, and the physical properties can be easily tuned between the gas and liquid which can quickly remove the heat released during the cyclization of PAN, improving the progress of the cyclization reaction. Furthermore, the aromatic cyclization first occurred in the amorphous region, the PAN molecular segment in the amorphous region was more likely to move in Sc-CO_2_, which was advantageous for the aromatic cyclization reaction. The aromatic cyclization reaction primarily caused an effective collision between the functional groups. The effective collisions formed among the functional groups in the macromolecular chains with a certain spatial position, a certain direction, and a certain angle. It required the molecular segments to have sufficient motion and soul to move, which could improve the opportunities for more effective collisions.

## 4. Conclusions

In this paper, Sc-CO_2_ as a new medium used in the pre-oxidation process of PAN fibers has been studied. FT-IR, DSC, XRD, TG, and density tests were performed to analyze the structural changes of PAN fibers during the cyclization reaction. The results indicated that the degree of cyclization increased with the increase of the cyclization temperature. Comparing the results of PAN fibers treated in two different media, the PAN fibers treated in Sc-CO_2_ possessed a higher degree of cyclization than those treated in the air from different test results. The density of the PAN fibers treated in Sc-CO_2_ was larger than that in the air.

## Figures and Tables

**Table 1:** Summary of the thermal conductivity data of supercritical carbon dioxide (Sc-CO_2_) and air in different studies.

Medium	Reference	T(K)	P(MPa)	λ (mW·m^−1^·K^−1^)
Sc-CO_2_	Johns et al. [28]	381.07–473.38	8.32–30.6	30.98–61.57
	Le Neindre et al. [29]	311.25–960.85	7.6–127.8	27.7–166.0
	Michels et al. [30]	313.2–348.29	8.437–209.68	30–194.81
	Le Neindre [31]	326.65–951.15	10–120	30.8–154.4
Air	Stephan et al. [32]	373.0–573.0	/	9.359–26.35

**Table 2:** Residual mass of the PAN fibers treated in Sc-CO_2_ and air.

Different Treatment Conditions	Residual Mass (%)/air	Residual Mass (%)/Sc-CO_2_
Untreated fibers	33.54	33.54
140 °C	34.78	43.32
160 °C	43.11	47.43
180 °C	44.43	55.21
200 °C	46.21	55.80
220 °C	51.86	62.07
240 °C	61.36	63.45
260 °C	61.59	66.34
